# Serum microRNAs as biomarkers for recurrence in melanoma

**DOI:** 10.1186/1479-5876-10-155

**Published:** 2012-08-02

**Authors:** Erica B Friedman, Shulian Shang, Eleazar Vega-Saenz de Miera, Jacob Ulrik Fog, Maria Wrang Teilum, Michelle W Ma, Russell S Berman, Richard L Shapiro, Anna C Pavlick, Eva Hernando, Adam Baker, Yongzhao Shao, Iman Osman

**Affiliations:** 1 Interdisciplinary Melanoma Cooperative Group, New York University School of Medicine, New York, NY, USA; 2 Department of Surgery, New York University School of Medicine, New York, NY, USA; 3 Division of Biostatistics, New York University School of Medicine, New York, NY, USA; 4 The Ronald O. Perelman Department of Dermatology, New York University School of Medicine, New York, NY, USA; 5 Exiqon A/S, 2950, Vedbaek, Denmark; 6 Department of Medicine, New York University School of Medicine, New York, NY, USA; 7 Department of Pathology, New York University School of Medicine, New York, NY, USA

**Keywords:** Melanoma, Serum microRNA, Prognostic biomarkers, Recurrence, Surveillance

## Abstract

**Background:**

Identification of melanoma patients at high risk for recurrence and monitoring for recurrence are critical for informed management decisions. We hypothesized that serum microRNAs (miRNAs) could provide prognostic information at the time of diagnosis unaccounted for by the current staging system and could be useful in detecting recurrence after resection.

**Methods:**

We screened 355 miRNAs in sera from 80 melanoma patients at primary diagnosis (discovery cohort) using a unique quantitative reverse transcription-PCR (qRT-PCR) panel. Cox proportional hazard models and Kaplan-Meier recurrence-free survival (RFS) curves were used to identify a miRNA signature with prognostic potential adjusting for stage. We then tested the miRNA signature in an independent cohort of 50 primary melanoma patients (validation cohort). Logistic regression analysis was performed to determine if the miRNA signature can determine risk of recurrence in both cohorts. Selected miRNAs were measured longitudinally in subsets of patients pre-/post-operatively and pre-/post-recurrence.

**Results:**

A signature of 5 miRNAs successfully classified melanoma patients into high and low recurrence risk groups with significant separation of RFS in both discovery and validation cohorts (p = 0.0036, p = 0.0093, respectively). Significant separation of RFS was maintained when a logistic model containing the same signature set was used to predict recurrence risk in both discovery and validation cohorts (p < 0.0001, p = 0.033, respectively). Longitudinal expression of 4 miRNAs in a subset of patients was dynamic, suggesting miRNAs can be associated with tumor burden.

**Conclusion:**

Our data demonstrate that serum miRNAs can improve accuracy in identifying primary melanoma patients with high recurrence risk and in monitoring melanoma tumor burden over time.

## Background

Melanoma remains a highly morbid disease in the United States, and its incidence has continued to rise over the past few decades [1]. The toll in terms of “life-years lost” in melanoma is the highest of all solid tumors in the United States [2]. Recurrence risk varies by stage, with estimated recurrence rates of up to 30% for localized melanoma and 60% for regional nodal disease [3]. Both the identification of patients at high risk for recurrence at primary diagnosis and the early detection of disease relapse are critical for informed management decisions.

The current standard of care for determining the prognosis of melanoma patients with localized disease and guiding post-operative follow-up both have limitations. In using prognostic factors, such as primary tumor thickness, ulceration, mitotic rate, and lymph node involvement, the American Joint Committee on Cancer (AJCC) staging system is able to provide first-line stratification for melanoma-specific survival [4]. However, the current staging system only partly explains the variability in the prognosis of melanoma, and there remains unexplained heterogeneity within each stage. Additionally, despite the benefit of early detection of locoregional and distant metastases amenable to curative resection [3,5], there is no consensus on either the selection or timing of imaging studies and laboratory tests for use in follow-up [6]. This is in part due to the limited sensitivity and specificity of available imaging modalities and blood tests, coupled with considerable economic cost [7].

Due to the routine collection and facility of obtaining blood samples at multiple time points, blood-based biomarkers are a logical and cost effective source in the search for non-invasive biomarkers. Although assessment of circulating markers for prognosis and surveillance has been part of the standard of care in breast and colon cancer management for several years [8], no such markers exist for early-stage melanoma. While many molecules have been studied as biomarkers for use in melanoma [9-11], none have been developed into a clinically relevant assay. Currently, serum lactate dehydrogenase is the only blood-based marker routinely used in melanoma, but it has only shown prognostic significance in advanced disease [4,10,11] and is rarely the sole indicator of recurrence [12]. There remains a need to develop methods to accurately determine recurrence risk of primary melanoma patients and improve follow-up for early detection of relapse.

MicroRNAs (miRNAs) are small, non-coding RNAs that negatively regulate gene expression at the post-transcriptional level [13]. Given their implication in tumorigenesis, tissue-specific dysregulation in cancer [14], and presence in human serum in an extremely stable form resistant to RNase digestion, harsh conditions, extended storage, and multiple freeze-thaw cycles [15], miRNAs have emerged as a novel source of blood-based reporters of cancer progression. Studies assessing serum or plasma miRNAs as biomarkers in a variety of cancers have largely focused on distinguishing cancer patients from control subjects [16,17]. While there is growing interest in evaluating the prognostic potential of circulating miRNAs [18-21], to the best of our knowledge, no study has specifically examined the association between serum-based miRNAs and recurrence risk in primary melanoma patients.

## Methods

### Study population

A total of 140 primary cutaneous melanoma patients who presented to New York University (NYU) Langone Medical Center for surgical resection of AJCC stage I-III disease were studied. Pre-treatment sera were collected at the time of initial diagnosis. A discovery cohort of 80 patients (discovery cohort) and an independent cohort of 50 patients (validation cohort) were used to construct prognostic models. Sera collected at the time of primary diagnosis and recurrence were available for a subset of patients (n = 17). All patients had histologically confirmed melanoma [22]. Relevant demographic and clinicopathological data were obtained from the prospectively maintained NYU Interdisciplinary Melanoma Cooperative Group (IMCG) database [22]. Both locoregional (stage III) and distant metastases (stage IV) were defined as recurrences. Additionally, an independent set of 10 melanoma patients had sera collected before and 7–14 days after surgical resection. The NYU Institutional Review Board approved the study and informed consent was obtained from all patients at the time of enrollment.

All serum samples were collected in 10-mL BD serum tubes, stored immediately at 4°C, and then centrifuged at 10°C for 10 minutes at 1,500 x g. The supernatant serum was then aliqouted into 1.5 mL cryovials and stored at −80°C until further use.

### miRNA extraction, quantitative reverse transcription-PCR (qRT-PCR) screening and individual evaluation

Total RNA was extracted from sera using the Qiagen miRNeasy® Mini Kit using manufacturer’s instructions with minor modifications and eluted with 50μL of RNase-free water. Reverse transcription (RT) was performed using 8 μl RNA in 40 μl reactions using the miRCURY LNA™ Universal RT microRNA PCR system (Exiqon, Denmark). cDNA was diluted 50x and amplified in 10 μl PCR reactions following manufacturer’s instructions by qPCR on a unique Pick & Mix discovery panel containing 355 miRNA assays and positive and negative controls. Negative controls excluding template from the RT reaction were performed and profiled like the samples. Amplification was performed in 384 well plates in a LightCycler® 480 Real-Time PCR System (Roche) and curves were analyzed using Roche LC software.

Notably, the qPCR platform (Exiqon) utilized has been reported to be the most sensitive and specific qPCR platform available when analyzing ultra low miRNA levels (200 copies or less) as found in serum and other biofluid samples [23]. The miRCURY platform has a very high degree of linearity (r^2^ ≥ 0.9) across four log scales of miRNA copies for all assays, indicating good performance of the panel at very low miRNA concentrations. True sensitivity of the platform is maximized because of two features. First, cDNA synthesis is carried out using a universal approach that allows all miRNAs in a sample to be synthesized equally. Second, LNAs are incorporated into the PCR primers making the PCR assay performance independent of GC content, which can otherwise affect the miRNAs ability to be efficiently amplified and accurately quantified.

Of the 355 miRNA assays, 170 miRNAs were included in analysis as they had Ct ≤ 40 and were detected with 5 Cts less than the no RT template included negative control in greater than two-thirds of patients. Expression data for 2 patients did not pass the quality control (QC) criteria and were not included in further analysis. Additionally, 1 patient had missing expression data for miR-199a-5p and was not included in analyses of models containing this miRNA.

A panel of 11 miRNAs (miR-15b, -23b, -30d, -33a, -103, -150, -199a-5p, -423-5p, -424, -425 and let-7d) identified as potential predictors of recurrence in multivariable models were selected for evaluation in the validation cohort using individual qRT-PCR assays. RT using 2 μl RNA in 10 μl reactions was performed in duplicate, amplified by qPCR and analyzed following the protocol described above.

### Longitudinal evaluation of miRNA expression

In exploring the potential of serum miRNAs as markers of melanoma recurrence, we elected to use a targeted approach to select miRNAs for study in pre-/post-operative and pre-/post-recurrence samples. We included several of the miRNAs that we identified as having prognostic potential in our discovery phase and were further evaluated in the validation cohort (i.e. miR-103, -191, -423-5p, -425). We did not limit ourselves to using only those miRNAs included in the recurrence risk signature as markers of prognosis may not translate into markers of disease detection. Instead, we focused on miRNAs from our prioritized panel that had previously supported roles in cancer progression and/or diagnostic utility. For example, high levels of miR-103 are associated with metastasis and poor outcome in breast cancer patients and functionally, mir-103 confers migratory capacities in vitro [24]. Elevated expression of miR-191 promotes epithelial-to-mesenchymal transition in hepatocellular carcinoma [25]. miR-423-5p was identified as part of a 5-miRNA signature for gastric cancer diagnosis [26]. We also chose to include one miRNA, miR-425, whose functional relevance in cancer was previously unexplored to avoid eliminating potentially useful markers. Additionally, we selected miRNAs that have well supported roles in melanoma progression (i.e. miR-182, -221, -222) as demonstrated by tissue-based studies [27,28].

Total RNA was extracted using the miRVana Paris isolation system (Ambion, USA) following the manufacturer’s protocol with the addition of an acid/phenol/chloroform extraction in a final elute of 100 μl. RT was performed using 2.5 μl miRNA in a final volume of 10 μl following manufacturer’s instructions using TaqMan MicroRNA Reverse Transcription kit (Applied Biosystems, USA). qRT-PCR was performed in triplicate on a MyIQ Single Color Real Time PCR detection system (Bio-Rad, USA), using 1.33 μl cDNA, 1 μl miRNA-specific TaqMan® primer and 1x Hotmaster Master mix containing Taq DNA polymerase (5 Prime, USA) and dNTP Mix (Promega, USA) in a final volume of 20 μl per reaction. The amplification protocol was: 95°C for 17 min, 40 cycles at 95°C for 15 sec followed by 60°C for 1 min.

### Statistical methods

Using the discovery cohort (n = 80), miRNAs were first ranked by univariate association of expression level for each miRNA with recurrence-free survival (RFS) via Cox proportional hazards regression analysis with adjustment for tumor stage. Top-ranking miRNAs were used as candidates to be included in the multivariate Cox proportional hazards model. The 5 miRNA-signature was selected by minimizing Akaike’s information criterion (AIC) of the multivariate Cox proportional hazards model through stepwise selection [29,30]. The linear combination of model predictors weighted by regression coefficients was defined as the risk score. A cutoff of the risk score was chosen to separate patients into high and low recurrence risk groups [31]. Kaplan-Meier survival curves for the resulting groups were plotted, and log-rank test was used to compare the two curves. To test the classifier, regression coefficients of the Cox model were applied to the validation cohort (n = 50) to obtain a risk score, and the same cutoff was used to predict RFS in the validation cohort.

The identified miRNA signature set was also evaluated for its utility in predicting 3-year RFS by logistic regression model using recurred patients (n = 25) and non-recurred patients (n = 44) with ≥3 years follow-up from the discovery cohort as cases and controls, respectively [32]. An optimal risk score cutoff using the Youden Index of the Receiver Operating Characteristic (ROC) curve was chosen to classify patients into high and low risk groups. Kaplan-Meier survival curves and log-rank tests were used to compare the RFS distributions of the two groups. The logistic model was used to predict recurrence risk in the validation cohort of 20 recurred and 16 non-recurred patients with ≥3 years follow-up. The same risk score cutoff was used to classify patients.

As a subset analysis to demonstrate the utility of serum miRNAs beyond tumor stage, we built a logistic model using the panel of 11 prioritized miRNAs in all stage II patients and examined its ability to distinguish patients with and without recurrence by cross validation, and examined the RFS difference using Kaplan-Meier curves between the predicted high vs. low risk groups. Longitudinal changes in miRNA expression were assessed using two-sided Student’s *t* tests (p < 0.05). All statistical analyses were performed in *R* 2.12.0.

The reported results are based on Ct values that passed QC but without further normalization. To assess the method for analysis, various ways of normalizing miRNA expression were explored (i.e. median normalization: shift Ct values on each panel by additive constants such that the medians of miRNA expression are the same across panels). The resulting candidate set of miRNAs identified were similar to those obtained when data were not normalized; moreover, selected classifiers worked well whether Ct values were normalized or not, indicating the robustness of the developed models.

## Results

Clinical and pathologic characteristics of the 130 patients used in prognostic modeling are presented in Table 1. Recurrence status stratified by stage for patients in the discovery and validation cohorts is shown in Table 2. The median time of follow-up for survivors was 42.5 months (range, 17–237 months).

**Table 1:** Baseline characteristics of melanoma patients

**Variable**	**Melanoma patients (n = 140)N (%)**
Age at diagnosis, years	
Median	59
Gender	
Male	82 (59)
Female	58 (41)
Thickness, mm	
Median (range)	2 (0.27-28)
Ulceration	
Present	49 (35)
Absent	91 (65)
Mitosis	
Absent	27 (19)
Present	97 (69)
Unclassified	16 (11)
Histological type	
Superficial Spreading	54 (39)
Nodular	44 (31)
Other^a^	25 (18)
Unclassified	17 (12)

NOTE. Percentages may not sum to 100% as a result of rounding

^a^Other includes acral lentiginous melanoma, desmoplastic melanoma and lentigo maligna.

**Table 2:** Recurrence status of melanoma patients stratified by stage

**Stage**	**Discovery Cohort**		**Validation Cohort**	
	**No recurrence**	**Recurrence**	**No recurrence**	**Recurrence**
I	34	5	10	0
II	13	7	16	12
III^a^	8	13	4	8
Totals	55	25	30	20

^a^Includes 1 patient diagnosed with stage III melanoma, but did not have blood drawn until time of recurrence with stage IV disease.

### Cox proportional hazards model identifies a miRNA signature with prognostic potential

**Figure 1:**
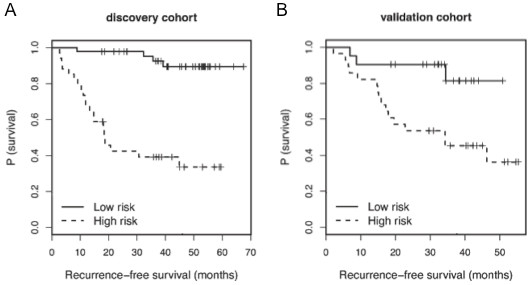
**Kaplan-Meier analysis for RFS by recurrence risks defined by the Cox proportional hazards model.** Patients defined as high recurrence risk (dashed line) by the Cox proportional hazards model demonstrated significantly reduced RFS compared to patients defined as low recurrence risk (solid line) in both the (**A**) discovery (p = 0.0036 by log rank test) and (**B**) validation (p = 0.009 by log rank test) cohorts

We screened serum samples of the discovery cohort using a qPCR platform of 355 miRNAs, of which data for 170 miRNAs were available for at least 67% samples. A multivariate Cox proportional hazards model for RFS was identified, which contains 5 miRNAs (miR-150, -15b, -199a-5p, -33a, -424) with adjustment for stage (Table 3). The linear combination of model predictors weighted by regression coefficients was defined as the risk score. Motivated by Satzger *et al.*[31]*,* a cutoff was chosen to separate patients into high and low recurrence risk groups aimed at maximizing the log-rank statistic (sensitivity = 0.84, specificity = 0.76; high risk, n = 34, low risk, n = 46). Kaplan-Meier analysis revealed that the two groups have a significant separation in RFS (p = 0.0036, Figure 1A). The model was applied to predict recurrence risk in the validation cohort, and the same cutoff was used to separate patients into high and low recurrence risk groups (sensitivity = 0.84, specificity = 0.60; high risk n = 28, low risk n = 21). Kaplan-Meier analysis indicated that the resulting RFS distributions were again significantly different (p = 0.009, Figure 1B). 

**Table 3:** Covariates included in multivariate Cox proportional hazards models

**Covariate**	**p-value**	**HR (95% CI)**
Stage II	0.0108	4.862 (1.442-16.397)
Stage III	4.1e-05	9.366 (3.125-27.287)
miR-150	0.1469	1.297 (0.913-1.843)
miR-15b	0.0159	0.437 (0.223-0.856)
miR-199a-5p	0.1383	1.375 (0.903-2.094)
miR-33a	0.1099	0.720 (0.481-1.077)
miR-424	0.0094	1.821 (1.158-2.862)

Abbreviation: HR: Hazard ratio; CI: confidence interval.

### Logistic regression analysis confirms prognostic potential of miRNA-based risk model for 3-year RFS

**Figure 2:**
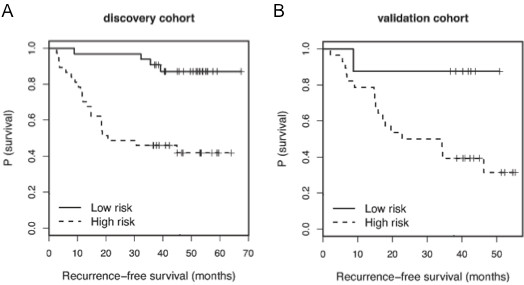
**Kaplan-Meier analysis for RFS by recurrence risks defined by logistic regression risk model.** Patients defined as high recurrence risk (dashed line) by the logistic regression model demonstrated significantly reduced RFS compared to patients defined as low recurrence risk (solid line) in both the (**A**) discovery (p < 0.0001 by log rank test) and (**B**) validation (p = 0.033 by log rank test) cohorts

The performance of the 5 miRNAs identified by Cox analysis was then evaluated by logistic regression analysis. In the discovery cohort, the addition of the 5-miRNA signature to a base risk model containing stage as a predictor improved the performance of the classifier, demonstrated by an increase in the area under the ROC curve (AUC) from AUC = 0.77 (95% CI, 0.66-0.89) of stage alone to AUC = 0.84 (95% CI, 0.73-0.94) with the 5-miRNA signature. Using a cutoff to separate the discovery cohort into high and low recurrence risk groups (sensitivity = 84%, specificity = 64%), the two groups had significant separation of RFS (p < 0.0001, Figure 2A). The model was applied to predict recurrence in the validation cohort, and the same cutoff was used to define high and low recurrence risk groups (sensitivity = 95%, specificity = 41%). Performance of the classifier was maintained, with significant separation of RFS (p = 0.033, Figure 2B).

### A signature of candidate miRNAs as predictors of recurrence in stage II melanoma supports that miRNA signatures might have prognostic utility beyond tumor stage

**Figure 3:**
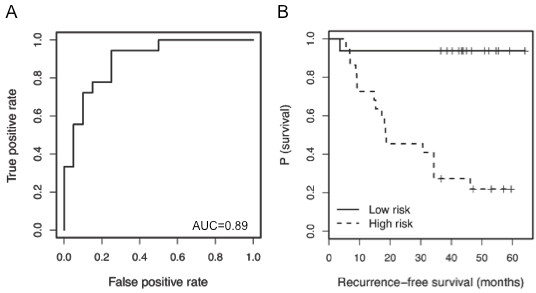
**Proof-of-principle logistic regression subgroup analysis of stage II patients.** (**A**) ROC curve for the miRNA containing logistic risk model defined for stage II patients had good classification performance (AUC = 0.89). (**B**) Kaplan-Meier analysis for RFS of high and low recurrence risk groups in stage II patients showed significant separation of RFS curves (p < 0.001 by log rank test)

Recurrence status among all stage II patients was relatively balanced, with 19 recurred and 20 non-recurred after ≥3 years follow up. Thus, this group was selected for subgroup analyses as proof-of-principle that serum-based miRNAs have prognostic potential beyond stage. Stage II patients pose a particularly difficult clinical challenge. Though still localized melanoma, stage II patients have significantly lower survival rates than stage I patients, with 5-year survival rates from 53% to 82% compared to >90%, respectively [33]. In our cohort of stage II patients, a logistic risk model with thickness alone was able to distinguish recurred from non-recurred patients with an AUC = 0.75 (p = 0.06). With the addition of 3 of the miRNAs prioritized for further evaluation (miR-423-5p, -424, -199a-5p), the performance of the classifier improved (AUC = 0.89, Figure 3A), and 2 of the miRNAs were significant predictors of recurrence in the model (miR-423-5p: Odds Ratio (OR), 0.038; 95% CI, 0.0031-0.46; p = 0.008; miR-424: OR, 3.64; 95% CI, 1.19-11.1; p = 0.018). Four-fold cross validation demonstrated an AUC = 0.81. Using Youden’s index of ROC curve as a cutoff to separate patients into high and low recurrence risk groups (sensitivity = 94%, specificity = 75%), the two groups had significant separation of RFS (p < 0.0001, Figure 3B). 

### miRNAs show potential in monitoring for melanoma recurrence

**Figure 4:**
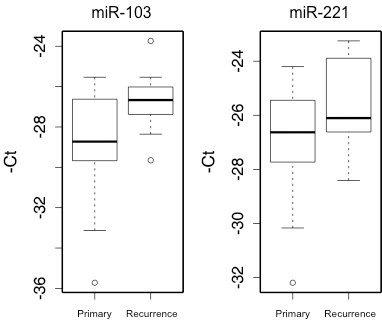
**Longitudinal evaluation of miRNA expression in pre- and post-recurrence serum samples.** Difference between miRNA expression levels of miR-103 and miR-221 at primary diagnosis and at recurrence was statistically significant (p = 0.012 and p = 0.026, respectively). The horizontal axis represents time of blood draw for 17 patients. The vertical axis represents –(Ct) value

To evaluate the clinical feasibility of using circulating miRNAs in post-resection surveillance for recurrence, we first measured the expression of 7 miRNAs, selected from our prioritized panel of 11 miRNAs based on previously supported roles in cancer progression and/or diagnostic utility and from tissue-based miRNA studies supporting an association with melanoma progression [27,28], in 10 matched serum samples collected pre- and post-resection of primary melanoma. Mean expression levels of all 7 miRNAs were reduced after operation, but only the difference in mean expression of miR-221, -222 and −423-5p reached statistical significance (p < 0.05). Next, we measured the expression of these miRNAs in pre- and post-recurrence serum samples from a subset of the discovery cohort (n = 17). Serum levels of all 7 miRNAs were increased, with mean expression of 2 miRNAs (miR-103 and −221) showing statistically significant post-recurrence elevation (p < 0.025, Figure 4). 

## Discussion

We present the first study demonstrating the potential prognostic value of serum miRNAs in melanoma. Specifically, we provide evidence that serum miRNAs show promise as biomarkers for improved identification of high-risk melanoma patients at the time of primary diagnosis. By doing so, we introduce biological subclassifiers that could add to the utility of clinical prognostic indicators and help account for latent heterogeneity in melanoma. This is increasingly important as better adjuvant therapies are developed, as it would enable clinicians to target those likely to recur for aggressive treatment.

We identify miRNAs that are potential markers of disease progression in melanoma patients. In other malignancies, such as colon, liver, testicular, prostate, and ovarian cancers, serum tumor markers have become part of the standard of care in surveillance for recurrence, at times used in lieu of periodic imaging in asymptomatic patients [34]. In melanoma, however, there are no widely available circulating markers that can facilitate early detection of relapse. Here, we show support for an association between serum miRNAs and tumor burden, a feature that if further developed, could be exploited by incorporation of serum miRNAs assays in routine surveillance for melanoma recurrence.

While there are several tissue-based and *in vitro* studies of miRNA dysregulation in melanoma [28,32,35-39], little is known about the “source” of serum miRNAs [19,21]. There is speculation that the source is not limited to the tumor and can include miRNAs shed from the tumor microenvironment or from circulating immune cells that also reflect the myriad of events involved in the initiation and development of malignancy. This suggests that, though tissue-based analyses are useful for understanding cancer biology, there is a complimentary role for studying circulating miRNAs as an independent source of biologically and clinically relevant information.

Growing interest in circulating miRNAs as biomarkers has resulted in several studies exploring their potential in solid tumors. However, the emphasis has been placed on elucidating the diagnostic capabilities of blood-based miRNAs [16,17]. In melanoma, one group reported on serum miR-221 levels and demonstrated a correlation with tumor thickness [40]. The second used array screening to identify unique miRNA expression profiles in peripheral blood cells of melanoma patients compared to healthy controls [35], which requires analysis of fresh blood specimens, and limits the practicality of the approach.

While we are the first to identify the prognostic relevance in melanoma of three of the miRNAs included in the recurrence risk signature (miR-199a-5p, -33a, -424), the biological function of all the identified miRNAs have begun to be elucidated in tissue and *in vitro* studies. miR-150 directly targets MUC4 in pancreatic cancer cells, an aberrantly overexpressed transmembrane mucin promoting growth, invasion and metastasis. miR-150 overexpression inhibits growth, clonogenicity, migration and invasion, and enhances intracellular adhesion in pancreatic cancer cells [41]. Clinically, miR-150 was elevated in melanoma tissues of patients with longer post-recurrence survival [39], also supporting a tumor suppressor role. Conflicting data exist regarding a pro-proliferative effect of ectopic expression of miR-150 in gastric cancer both in vitro and in vivo, at least in part due to repression of the tumor suppressor EGR2, resulting in translational arrest [42]. Our findings are in line with an oncogenic role for miR-150, with higher circulating expression of miR-150 in patients with a high recurrence risk. However, our findings can also be due to the possibility that serum miRNAs reflect the systemic immune response as miR-150 has a role in the modulation of the T-cell development. Specifically, via modulation of NOTCH3, overexpression of miR-150 has adverse effects on T-cell proliferation and survival, resulting in decreased antitumor immunity and subsequent progression [43].

In our model, patients with high recurrence risk had lower levels of miR-15b, similar to findings in a recent study showing an association between reduced miR-15b expression, chemotherapeutic resistance and poor prognosis in patients with tongue squamous cell carcinoma [44]. The same study identified BIM1 as a functional target of miR-15b, through which BIM1 overexpression due to reduction of miR-15b regulates epithelial to mesenchymal transition and chemoresistence. Also in line for a tumor suppressor role for miR-15b is its direct regulation of the critical anti-apoptotic Bcl-2 protein [45]. On the other hand, a previous study in melanoma demonstrated that miR-15b downregulation in miR-15b high melanoma cell lines resulted in decreased proliferation and increased apoptosis and clinically, found increased tissue-based expression of miR-15b in melanoma FFPE samples from patients with shorter recurrence-free and overall survival [31]. However, these differences may be attributable to host-specific effects reflected by measurement of circulating miRNAs as opposed to solely tumor-specific effects.

While we are the first to demonstrate the prognostic potential of miR-199a-5p in melanoma, with higher expression in high recurrence risk patients, its diagnostic potential was supported by a study that found miR-199a-5p overexpression in blood cells of melanoma patients compared to healthy controls [35]. Our data, taken with a recent study that found miR-199a-5p was one of several miRNAs overexpressed in a small sample of brain-metastatic compared to primary colorectal carcinomas [46], invites speculation that miR-199a-5p has a role in the process of dissemination beyond its role in carcinogenesis, which could in part be due to modulation of Brm-type SWI/SNF activity [47]. Future studies will be needed to further define the role of this miRNA in cancer metastasis, and specifically in melanoma.

Strong evidence exists supporting a tumor suppressor function for miR-33a via repression of proto-oncogene Pim-1 [48] and inhibition of expression of cyclin-dependent kinase 6 (CDK6) and cyclin D1 (CCDN1) [49], both of which results in reduced cellular proliferation and cell-cycle progression. Though not previously studied in melanoma, our data is also suggestive of a tumor suppressive function for miR-33a, with lower levels of miR-33a associated with high recurrence risk scores. As with miR-33a, the prognostic or functional relevance of miR-424 has not been studied in melanoma, but roles have been previously defined for miR-424 in HIF-1α/HIF-2α mediated angiogenesis [50] and regulation of monocyte/macrophage differentiation [51]. Given these roles of miR-424 in tumor formation and dissemination (i.e. angiogenesis) and in systemic immune responses, it is not surprising that, in measuring circulating miRNAs, we found patients with high recurrence risk to have elevated levels of miR-424 in the recurrence risk model. Though the biological role and mechanistic relationship of each of these components are not completely understood, particularly as they relate to melanoma, these miRNA prognostic classifiers can still be clinically useful [52].

The significant post-operative reduction and subsequent elevation at recurrence of the miRNAs identified as having potential in detection of disease relapse (miR-103, -221, -222, -423-5p) is consistent with existing data delineating their roles in cancer progression. miR-103, via downregulation of the enzyme Dicer, promotes cell migration and invasion in breast cancer cells in vitro [24]. Clinically, high levels of miR-103 are associated with metastasis and poor outcome in breast cancer patients [24]. *In vitro* and *in vivo* studies support oncogenic roles for miR-221/222, which function to increase invasion and migration capabilities as well as proliferative growth rate in melanoma by targeting of c-kit, p27, and p57 [28]. Though the functional relevance and target genes of miR-423-5p have yet to be uncovered, serum levels were significantly higher in gastric cancer patients compared to healthy controls and it was identified as part of 5-miRNA signature for gastric cancer diagnosis [26]. Together with our results that demonstrate higher levels at the time of recurrence, we can begin to speculate about a possible oncogenic role for miR-432-5p.

We acknowledge that our study has several limitations. First, we performed unbiased serum miRNA expression profiling using a system demonstrated to have the highest sensitivity for analyzing serum miRNAs [23]. With 1,500+ transcribed miRNAs identified in the human genome [16], the use of a panel containing 355 miRNA assays could potentially be limiting. However, the miRNAs included were identified as being a comprehensive set of miRNAs expressed in serum from extensive genome wide qPCR screening of normal and diseased samples, including melanoma patients [23]. Next, given the expected imbalance in recurrence status among stage I and III patients, we cannot speculate about a predictive model that would work across all stages. However, we are able to prioritize a manageable panel of miRNAs for future testing that show potential as circulating biomarkers with clinical relevance in identifying primary melanoma patients at high risk for recurrence.

## Conclusion

The evolving paradigm shift towards a molecular characterization of melanoma to improve prognostic accuracy, detect recurrence, and better guide management decisions has largely been devoid of blood-based miRNA studies. We show that serum-based miRNAs demonstrate hallmarks of useful tumor markers, namely easy detection in accessible samples and promising clinical utility and applicability in melanoma. We demonstrate support for the use of serum miRNAs as clinically useful non-invasive biomarkers that could potentially enhance the utility of current prognostic factors in predictive models of melanoma recurrence. This would allow for improved stratification of a heterogeneous cancer that could be influential in clinical decision-making. Specifically, the development of serum miRNAs into clinical assays can refine criteria for more extensive staging procedures, such as sentinel lymph node biopsy, and for inclusion in clinical trials. Moreover, the potential exists for serum miRNAs to not only guide follow-up recommendations and allow for better allocation of resources, but also to improve the power of the current arsenal of imaging and laboratory tests used in routine surveillance. Future large prospective studies focusing on the identified serum-based miRNAs stand to have a potentially large clinical impact in aiding the early identification of primary melanoma patients with high risk for recurrence and in the timely detection of disease relapse.

## Abbreviations

miRNAs: microRNAs; qRT-PCR: Quantitative reverse transcription-PCR; RFS: Recurrence-free survival; AJCC: American Joint Committee on Cancer; NYU: New York University; IMCG: Interdisciplinary Melanoma Cooperative Group; RT: Reverse transcription; QC: Quality control; AIC: Akaike’s Information Criterion; ROC: Receiver Operating Characteristic; AUC: Area under the curve; OR: Odds ratio; CI: Confidence interval.

## Competing interests

MWT and AB are currently paid employees of Exiqon A/S, Denmark. JUF was a paid employee of Exiqon A/S, Denmark at the time the research was conducted.

## Authors’ contributions

EBF participated in study design, coordination, data collection, data analysis, drafting, revising and finalizing of the manuscript. SS performed the statistical design and analysis under the guidance of YS and participated in the writing of the manuscript. EV participated in study design and data collection. JUF participated in study design and data collection. MWT participated in study design and data collection. MWM participated in data analysis and manuscript drafting/finalization. RSB recruited patients for the study, provided input on study design, and helped to draft the manuscript. RLS recruited patients for the study, provided input on study design, and helped to draft the manuscript. ACP recruited patients for the study, provided input on study design, and helped to draft the manuscript. EH participated in the conceptual study design, provided guidance regarding interpretation of results, and helped with manuscript preparation and finalization. AB provided guidance in study design, data collection, and writing of the manuscript. YS was the principal statistician for the study, providing guidance on statistical design and data analysis. IO served as the principal investigator for the project, overseeing the study design, analysis of data, interpretation of results, and writing of the manuscript. All authors read and approved the final manuscript.
